# Next-generation genome annotation: we still struggle to get it right

**DOI:** 10.1186/s13059-019-1715-2

**Published:** 2019-05-16

**Authors:** Steven L. Salzberg

**Affiliations:** Departments of Biomedical Engineering, Computer Science, and Biostatistics, Johns Hopkins University, Baltimore, MD 21205 USA

## Abstract

While the genome sequencing revolution has led to the sequencing and assembly of many thousands of new genomes, genome annotation still uses very nearly the same technology that we have used for the past two decades. The sheer number of genomes necessitates the use of fully automated procedures for annotation, but errors in annotation are just as prevalent as they were in the past, if not more so. How are we to solve this growing problem?

## Introduction

When the first complete bacterial genome, *Haemophilus influenzae*, appeared in 1995, the 1.83 megabase (Mb) sequence was accompanied by annotation of 1742 protein-coding genes along with a small complement of transfer RNAs (tRNAs) and ribosomal RNAs [1]. This genome paper, and the dozen or so that followed in the next few years, defined genome annotation as it still exists today: the process of decorating the genome with information about where the genes are and what those genes (might) do. Over the years, efforts to expand the scope of annotation have flourished, and today we have information about a wide range of other functional elements, including noncoding RNAs, promoter and enhancer sequences, DNA methylation sites, and more. Nonetheless, the core feature of genome annotation is still the gene list, particularly the protein-coding genes. With hundreds of eukaryotic genomes and well over 100,000 bacterial genomes now residing in GenBank, and many thousands more soon to come, annotation is a critical element to help us understand the biology of genomes.

Paradoxically, the incredibly rapid improvements in genome sequencing technology have made genome annotation less, not more, accurate. The main challenges can be divided into two categories: (i) automated annotation of large, fragmented “draft” genomes remains very difficult, and (ii) errors and contamination in draft assemblies lead to errors in annotation that tend to propagate across species. Thus, the more “draft” genomes we produce, the more errors we create and propagate. Fortunately, technological advances give us some hope that we can mitigate these problems, even if a full solution is still beyond our reach.

## High-throughput annotation of eukaryotic genomes

Finding genes in bacteria is relatively easy, in large part because bacterial genomes are approximately 90% protein-coding, with relatively short intergenic stretches in between every pair of genes. The gene-finding problem is mostly about deciding which of the six possible reading frames (three in each direction) contains the protein, and computational gene finders take advantage of this to produce highly accurate results. Thus, although we still don’t know the functions of many bacterial genes, at least we can be confident that we have their amino acid sequences correct.

In eukaryotes, by contrast, the gene-finding problem is far more difficult, because (i) genes are few and far between, and (ii) genes are interrupted by introns. Thus, while 90% of a typical bacterial genome is covered by protein-coding sequences, only about 1.3% of the human genome (40.2 Mb in the CHESS 2.2 database [2]) comprises protein-coding exons. The percentage is even lower in larger genomes, such as the mega-genomes of pine trees and other conifers. For this reason and others, the best automated gene finders are far less accurate on eukaryotes. Manual curation will not solve this quandary, for the obvious reason that it does not scale, and the less-obvious reason that even careful human analysis does not always provide a clear answer. To illustrate the latter point: in a recent comparison of all the protein-coding and lncRNA transcripts in the RefSeq and Gencode human gene databases, only 27.5% of the Gencode transcripts had exactly the same introns as the corresponding RefSeq genes [2]. Thus, even after 18 years of effort, the precise exon–intron structure of many human protein-coding genes is not settled. The annotation of most other eukaryotes—with the exception of small, intensively studied model organisms like yeast, fruit fly and *Arabidopsis*—is in worse shape than human annotation.

One high-throughput solution provides at least a partial solution to this problem: RNA sequencing (RNA-seq). Prior to the invention of RNA-seq, scientists worked hard to generate full-length transcripts that could provide a “gold standard” annotation for a species. The idea was that if we had the full-length messenger RNA sequence for a gene, we could simply align it to the genome to reveal the gene’s exon–intron structure. The Mammalian Gene Collection, an effort to obtain these RNAs for humans and a few other species, concluded in 2009 with the announcement that 92% of human protein-coding genes had been captured [3]. That project, though extremely useful, was very expensive, not easily scalable, and still not comprehensive. (Notably, the Mammalian Gene Collection only attempted to capture a single isoform of each gene. We now know that most human genes have multiple isoforms.) RNA-seq technology, in contrast, provides a rapid way to capture most of the expressed genes for any species. By aligning RNA-seq reads to a genome and then assembling those reads, we can construct a reasonably good approximation (including alternative isoforms) of the complete gene content of a species, as my colleagues and I have done for the human genome [2].

Thus, a modern annotation pipeline such as MAKER [4] can use RNA-seq data, combined with alignments to databases of known proteins and other inputs, to do a passably good job of finding all genes and even assigning names to many of them.

This solution comes with several major caveats. First, RNA-seq does not precisely capture all of the genes in a genome. Some genes are expressed at low levels or in only a few tissues, and they might be missed entirely unless the RNA sequencing data are truly comprehensive. In addition, many of the transcripts expressed in a tissue sample are not genes: they might represent incompletely spliced transcripts, or they might simply be noise. Therefore, we need independent verification before we can be certain that any expressed region is a functional gene. Even for genes that are repeatedly expressed at high levels, determining whether they encode proteins or instead represent noncoding RNAs is a still-unsolved problem. The current Gencode human annotation (version 30), for example, contains more RNA genes than proteins [5], but no one knows what most of those RNA genes do.

Another caveat is that because draft genomes may contain thousands of disconnected contigs, many genes will be broken up among several contigs (or scaffolds) whose order and orientation are unknown. The problem occurs in all species, but it is much worse for draft genomes where the average contig size is smaller than the span of a typical gene. This makes it virtually impossible for annotation software to put genes together correctly; instead, the software will tend to annotate many gene fragments (residing on different contigs) with the same descriptions, and the total gene count might be vastly overinflated. Even where they don’t have gaps, some draft genomes have high error rates that may introduce erroneous stop codons or frame shifts in the middle of genes. There is no way that annotation software can easily fix these problems: the only solution is to improve the assemblies and re-annotate.

## Errors in assembly cause errors in annotation

Sequencing a bacterial genome or a small eukaryote is so fast and inexpensive today that a relatively small lab can easily afford the cost of deep whole-genome shotgun sequencing. After generating 100-fold coverage in 100–150 bp Illumina reads, a scientist can assemble the data into a draft genome using any of several genome assemblers. Ironically, though, the ease of sequencing and assembly presents another challenge for annotation: contamination of the assembly itself.

When a genome is assembled into thousands of contigs, the person doing the assembly has no easy way to ensure that every one of those contigs truly represents the target species. In some recent projects, draft genomes contained hundreds of contigs from foreign species; e.g., the tardigrade genome, which was sequenced from DNA collected from multiple whole animals. (This was a necessary step because a single tardigrade does not yield sufficient DNA for whole-genome sequencing.) The first publication of the tardigrade erroneously claimed that its contaminants represented an astounding number of horizontal gene transfer events; fortunately, a much better assembly was published very soon after the first one, in which the contaminants were identified and removed [6]. Other draft genomes have yielded similar claims of horizontal gene transfer, many of which are false positives due to contamination [7]. And many draft genome assemblies are contaminated with common bacteria [8], sequencing vectors, or even human DNA [9], all of which are ubiquitous presences in sequencing labs.

Although automated annotation is essential to keep pace with the vast number of new genomes, any error in existing annotation—whether it be a mistaken gene name, or a gene labeled as belonging to the wrong species, or a non-genic sequence being called a gene—is likely to be quickly propagated to other species. This presents one more (and growing) annotation challenge: when an annotation error is found and corrected in one species, any other annotation that relied upon it needs to be corrected as well. Currently there is no way to achieve this; indeed, public annotation databases do not record the source of every gene assignment.

## Coming soon: direct RNA sequencing

Finally, a newly emerging technology, direct sequencing of RNA [10], offers the possibility of dramatically improving gene annotation in the future. Although still in early development, nanopore sequencing technology can been used to sequence RNA without first converting it to DNA, unlike RNA-seq and other methods. With direct RNA sequencing, we may soon have the ability to generate full-length transcripts in a truly high-throughput manner, replacing years-long efforts of the past [3] with a rapid, low-cost solution that will be within the reach of many individual scientific labs. This approach, although not a panacea, promises to greatly improve our ability to describe the full complement of genes for every species.

## Abbreviation

RNA-seq: RNA sequencing
